# Digitally-supported patient-centered asynchronous outpatient follow-up in rheumatoid arthritis - an explorative qualitative study

**DOI:** 10.1186/s12913-022-08619-6

**Published:** 2022-10-28

**Authors:** Ramona Stenzel, Katharina Hadaschik, Susann May, Manuel Grahammer, Hannah Labinsky, Martin Welcker, Johannes Hornig, Gerlinde Bendzuck, Corinna Elling-Audersch, Ulrike Erstling, Patricia Steffens Korbanka, Nicolas Vuillerme, Martin Heinze, Gerhard Krönke, Georg Schett, Ann-Christin Pecher, Martin Krusche, Johanna Mucke, Johannes Knitza, Felix Muehlensiepen

**Affiliations:** 1 Department of Internal Medicine 3, Rheumatology and Immunology Friedrich Alexander University Erlangen-Nürnberg and Universitätsklinikum Erlangen, Erlangen, Germany; 2 Deutsches Zentrum für Immuntherapie, Friedrich-Alexander University Erlangen-Nürnberg and Universitätsklinikum Erlangen, Erlangen, Germany; 3 Center for Health Services Research, Faculty of Health Sciences Brandenburg, Brandenburg Medical School Theodor Fontane, Seebad 82/83, Rüdersdorf bei Berlin, Germany; 4 Abaton GmbH, Berlin, Germany; 5 MVZ für Rheumatologie Dr. Martin Welcker GmbH, Planegg, Germany; 6 Rheumapraxis an der Hase, Osnabrück, Germany; 7 Deutsche Rheuma-Liga Bundesverband e.V, Bonn, Germany; 8 Fachverband Rheumatologische Fachassistenz e. V, Bergisch Gladbach, Germany; 9 AGEIS, Université Grenoble Alpes, Grenoble, France; 10 Institut Universitaire de France, Paris, France; 11 LabCom Telecom4Health, Orange Labs & Univ. Grenoble Alpes, CNRS, Grenoble INP-UGA, Grenoble, Inria France; 12 Centre for Interdisciplinary Clinical Immunology, Rheumatology and Autoinflammatory Diseases, University Hospital Tuebingen, Tübingen, Germany; 13 Division of Rheumatology and Systemic Inflammatory Diseases, University Hospital Hamburg-Eppendorf (UKE), Hamburg, Germany; 14 Policlinic and Hiller Research Unit for Rheumatology, Medical Faculty, University Hospital Düsseldorf, Heinrich Heine University Düsseldorf, Düsseldorf, Germany

**Keywords:** Telemedicine, Rheumatology, User experience, Remote care, Qualitative research, Empowerment, Self-sampling, PIFU

## Abstract

**Objective:**

A steadily increasing demand and decreasing number of rheumatologists push current rheumatology care to its limits. Long travel times and poor accessibility of rheumatologists present particular challenges for patients. Need-adapted, digitally supported, patient-centered and flexible models of care could contribute to maintaining high-quality patient care. This qualitative study was embedded in a randomized controlled trial (TELERA) investigating a new model of care consisting of the use of a medical app for ePRO (electronic patient-reported outcomes), a self-administered CRP (C-reactive protein) test, and joint self-examination in rheumatoid arthritis (RA) patients. The qualitative study aimed to explore experiences of RA patients and rheumatology staff regarding (1) current care and (2) the new care model.

**Methods:**

The study included qualitative interviews with RA patients (n = 15), a focus group with patient representatives (n = 1), rheumatology nurses (n = 2), ambulatory rheumatologists (n = 2) and hospital-based rheumatologists (n = 3). Data was analyzed by qualitative content analysis.

**Results:**

Participants described current follow-up care as burdensome. Patients in remission have to travel long distances. Despite pre-scheduled visits physicians lack questionnaire results and laboratory results to make informed shared decisions during face-to-face visits. Patients reported that using all study components (medical app for ePRO, self-performed CRP test and joint self-examination) was easy and helped them to better assess their disease condition. Parts of the validated questionnaire used in the trial (routine assessment of patient index data 3; RAPID3) seemed outdated or not clear enough for many patients. Patients wanted to be automatically contacted in case of abnormalities or at least have an app feature to request a call-back or chat. Financial and psychological barriers were identified among rheumatologists preventing them to stop automatically scheduling new appointments for patients in remission. Rheumatology nurses pointed to the potential lack of personal contact, which may limit the holistic care of RA-patients.

**Conclusion:**

The new care model enables more patient autonomy, allowing patients more control and flexibility at the same time. All components were well accepted and easy to carry out for patients. To ensure success, the model needs to be more responsive and allow seamless integration of education material.

**Trial registration:**

The study was prospectively registered on 2021/04/09 at the German Registry for Clinical Trials (DRKS00024928).

**Supplementary Information:**

The online version contains supplementary material available at 10.1186/s12913-022-08619-6.

## Introduction

Rheumatoid arthritis (RA) is a chronic inflammatory autoimmune disease that requires lifelong medical support [1]. The disease is characterized by an individual disease fluctuation of disease activity with phases of remission and disease flares. International societies advocate a proactive “treat-to-target” strategy, continuous monitoring of disease activity and shared-decision-making (SDM) between patients and medical staff [2]. The dramatic workforce shortage and an increasing number of patients stress current rheumatology care. In clinical reality, patient management often seems reactive and inflexible, as patient visits are planned irrespective of disease activity and often do not cause treatment changes, due to an increased proportion of patients being in disease remission. Due to a lack of usage of electronic patient-reported outcomes by rheumatologists [3], the current monitoring approach is not continuous and often limited to a snapshot-approach of very limited physician visits [4]. Disease flares often occur in between pre-scheduled visits, are not monitored and are therefore not included in the treatment decision. The poor documentation further limits SDM, which leaves room for improvement [5]. To enable optimal SDM and treatment, patients need to become experts of their disease and receive adequate structured education [6]. Due to the limited available time for medical staff with the patients, this need for information is currently not met in clinical routine [7–9].

The European Alliance of Associations for Rheumatology (EULAR) recently acknowledged the potential of remote care and patient self-management to improve rheumatology care, and published respective recommendations [10]. Similarly, Brkic et al. previously addressed the urgent need for a decentralized digitally-supported follow-up of RA patients [11]. Shaw et al. were able to show the improvement of the patient–provider interactions by the integration of ePRO (electronic patient-reported outcomes) into the clinical care process [12]. Patient-initiated follow-up (PIFU) reduces medical visits to on-demand patient-initiated visits. In a landmark study, Fredriksson et al. showed that PIFU is as effective as traditional pre-scheduled appointments in early [13] and established RA [14]. In another important study, de Thurah et al. demonstrated that a tele-health follow-up strategy (pre-scheduled monitoring visits) enables the reduction of outpatient clinic visits without compromising quality and safety [15]. Furthermore, recently, it has been demonstrated that patients can also reliably self-collect blood for CRP (C-reactive protein) analysis, enabling the addition of objective laboratory parameters to remote care [16–18].

**Fig. 1:**
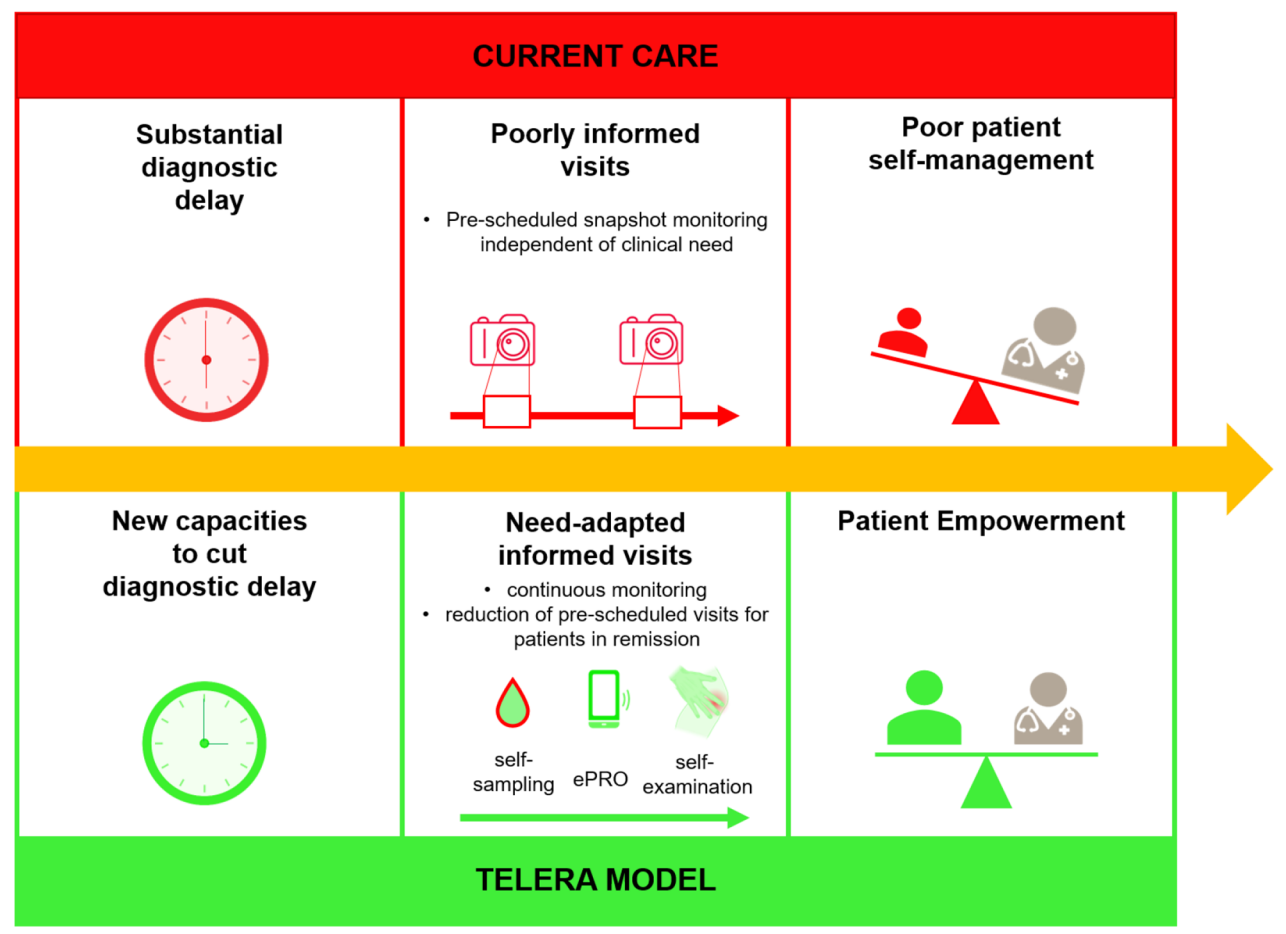
Current patient follow-up and investigated new care-model

Therefore, we were eager to explore the potential of a new decentralized digitally-supported patient-centered asynchronous RA care-model, and initiated the multicenter TELERA trial [19] (Fig. 1). In this trial RA Patients use a medical app, ABATON RA, to document electronic patient-reported outcomes (ePRO) in between visits. Additionally, patients have access to an instructional video to inform joint self-examination. Patients then perform a self-examination of joints, a capillary point-of-care semi-quantitative CRP test and complete ePRO, so that they and rheumatologists have access to an Auto-DAS28-CRP. Primary outcome of the trial is the agreement of therapeutic suggestions of patients and tele-rheumatologists compared to the gold-standard, the therapeutic decision by the local rheumatologist.

The aim of this qualitative study was to explore experiences of RA patients and medical staff regarding challenges in current standard RA follow-up, the new care-model and their perspective towards its transferability to standard RA care. The study was designed in close cooperation with official patient research partners of the German League against Rheumatism (Deutsche Rheuma-Liga Bundesverband e.V.)

## Methods

To explore the user perspectives towards the new care-model, we conducted qualitative interviews with RA-patients (n = 15) that had participated at one clinical site in the TELERA trial and a multiperspective focus group discussion with patient-representatives (n = 1), rheumatologists (n = 5) and specialised rheumatology nurses (n = 2).

Interview partners were identified using purposive sampling [20] with the aim to include a heterogeneous sample and thus cover diverse experiences and preferences towards follow-up in RA care. Inclusion criteria for patients were (1) the established diagnosis of RA according to the 2010 ACR/EULAR classification criteria; (2) > 18 years of age; (3) sufficient German language skills; (4) possession of and confidence in using a smartphone; and (5) written informed consent. As participants completed the trial, those who agreed were contacted by the research team to participate in the additional qualitative study and additional written consent was obtained. The interviews were conducted using an interview guide (Supplemental Material 1) that was developed to specifically elicit the participants’ experiences. The interview guide was developed by two health service researchers (F.M., S.M.), one rheumatologist (J.K.) and previous discussions with two patient research partners (G.B., C.E.A.). The interview guide included the following main topics: (1) Challenges in standard RA follow-up care, (2) Feasibility of the new care model, (3) Opportunities and barriers, and (4) Transferability to standard care. Initial exploratory questions were then specified by follow-up questions. We conducted pilot interviews to test and refine the interview guide. No revisions were necessary. Additional sociodemographic data were collected, including gender, age, diagnosis, education and profession. In order to reduce the risk of infection and lower patient burden, the interviews were conducted via telephone. The phone interviews took place in December 2021 and January 2022.

The focus group [21] was held in March 2022 on the topic “Flexible outpatient follow-up in rheumatoid arthritis”. In order to reduce the risk of infection, the focus group was conducted via videoconference (Cisco Webex Meeting). The purpose of the focus group was to contrast the perspectives of patient representatives, nurses and rheumatologists from different rheumatology care settings. The discussion was divided into three thematic sections: (1) Challenges in the follow-up of RA patients in standard care; (2) Asynchronous follow-up of RA patients: Opportunities and barriers; (3) Transferability to standard care. The discussion was stimulated by a synchronous documentation of the discussion content. The interviews and focus group were audio-recorded and transcribed verbatim.

Data collection and analysis were conducted simultaneously by three researchers (F.M., S.M., K.H.), based on Kuckartz’s structured qualitative content analysis [22] using MAXQDA software (Verbi GmbH). Categories were developed inductively, to encompass the relevant material in the transcripts using data-driven development of a coding tree Supplemental Material 2). Next, the category system was applied to the entire qualitative data. At this stage, data collection had already been completed. To ensure traceability, the application of the category system was validated by a member check, whereby the researchers independently applied the developed category system to the entire material (S.M., F.M.). Data collection and analysis were circular and continued until no substantially new findings emerged and theoretical saturation was reached.

The study has received ethical approval by the Ethical Committee of the University Clinic of Tuebingen (# 4442020BO) and was prospectively registered on 2021/04/09 at the German Registry for Clinical Trials (DRKS00024928). This manuscript has been compiled in accordance with the Consolidated Criteria for Reporting Qualitative Research (COREQ) (Supplemental Material 3) [23]. For the presentation of the results, representative quotes of the transcripts were selected, translated into English and included in the manuscript.

## Results

### Participant characteristics

From December 2021 to January 2022, we conducted qualitative interviews with RA-patients (n = 15), involved in the TELERA trial. The mean duration of the interviews was 17 (range: 8–30) minutes. Mean age of interviewed patients was 55 (range: 32–69) years. Most interviewed patients were female (12/15; 80%). Patients received their RA diagnosis on average 7 (range: 1–28) years ago. Patients reported diverse occupational and educational backgrounds **(**Table 1**).** On March 21st, 2022, we conducted a focus group with patient representatives (n = 1), rheumatology nurses (n = 2) as well as ambulatory rheumatologists (n = 2) and hospital-based rheumatologists (n = 3). The focus group duration was 68 min. Mean age of the focus group participants was 44 (range: 30–57) years. 56% of participants were female **(**Table 2**)**.

**Table 1:** Qualitative Interviews: Participants Characteristics

Patient	Age	Gender	Years since diagnosis	Education	Occupation
1	43	F	1	University Degree	Janitor
2	52	F	1	University Degree	Business economist
3	64	M	3	University Degree	Mechanical engineer
4	68	M	17	Middle School Degree	Retiree
5	48	F	5	High School Degree	Insurance employee
6	41	F	14	High School Degree	Retiree
7	50	F	2	Middle School Degree	Office worker
11	59	F	28	Middle School Degree	Retiree
9	69	F	3	Middle School Degree	Retiree
10	55	F	14	High School Degree	Accountant
11	56	F	7	Middle School Degree	Pharmaceutical commercial employee
12	63	F	2	Middle School Degree	Office Administrator
13	67	F	3	University Degree	Retiree
14	32	F	4	University Degree	Physician
15	64	M	6	University Degree	Retiree

**Table 2:** Focus Groups: Participants Characteristics

Participant	Age	Gender	Role
1	54	F	Patient Representative
2	54	F	Rheumatology Nurse
3	52	F	Rheumatology Nurse
4	37	M	Rheumatologist (ambulatory)
5	32	F	Rheumatologist (hospital)
6	57	M	Rheumatologist (ambulatory)
7	35	M	Rheumatologist (hospital)
8	30	M	Rheumatologist (hospital)

### Themes

The analysis revealed three key themes: (i) Standard RA follow-up care; (ii) TELERA trial user experiences; (iii) transfer to standard rheumatology care.

### Current challenges in the follow-up of RA

Specifically, during the focus group, challenges in rheumatologic care were discussed extensively. The study participants homogeniously reported the enormous workforce shortage as the main challenge in rheumatology care. This applies to both rheumatologists and specialist nurses, respectively. The shortage is particularly evident in rural areas. There are too few appointments for too many patients, with patient and appointment management procedures not yet fully optimized. New patients have to face long waiting times before receiving an appointment, causing irreversible damage that could be avoided; while well-adjusted RA-patients might be over-served and patients suffering short-term episodes cannot be treated quickly enough.

“We still have a situation where, depending on the region- In rural areas, two-thirds are under-served anyway, even according to the newly changed demand planning. In the urban area, perhaps only half is under-served - And the whole thing also in a not yet optimized patient management in such a way that well-adjusted rheumatism patients like me are so to speak over-served and not even necessarily all have to go to the rheumatologist once every quarter, which is why difficult cases somehow have to wait much, much too long for a first appointment. Then the first damage has already occurred, perhaps also as a result of this the first incapacities to work, somehow perpetuated.“ (Focus group, Patient Rep. 03:51).

According to the study participants, the current rheumatology care delivery is not need-driven, meaning that patients who need extra care should be prioritized. Moreover, the participants reported that too much HCP-time is spent on non-medical activities, simultaneously leaving too little time for patient education. Furthermore, workflows in rheumatology care are not structured efficiently, which leads to not having all relevant information available in the consultation:

“The sequence of diagnostic findings, for example, that we see the patients, then do the laboratory and, if there are any abnormalities, contact the patient again and perhaps have to completely change what we discussed, because it would perhaps make more sense to take the laboratory beforehand, then use it to come to the appointment and also replace the paper questionnaires with electronic ones in order to be able to do something with the data more quickly, in order to perhaps also record them beforehand, to go into the appointment much more informed, to better record how the patients are doing in between appointments.” (Focus group, rheumatologist 07:43).

Interviewed patients described predominantly the journeys to the rheumatologist as burdensome:

“For me, that’s always 120 kilometers there and back. And now, with the fuel prices.” (P4, 07:00).

“The distance wasn’t the problem either, but rather finding a parking space.“ (P6, 09:00).

### TELERA trial user experiences

In the interviews, patients were asked about their experiences in the study. Most interviews initially reported their experiences with the app:

*Interviewer:* And what did you do in the trial? Please describe.

*Interviewee*: I answered questions weekly on this app about how I was doing and if I was having relapses. Yes, there were two blocks of questions, I answered them weekly. (P3, 02:29)

Whereas independent self-sampling and joint-examination were reported as relatively little change compared to standard routine.

*Interviewer*: All right then. Another question: The palpation of your joints, how was that? Could you please describe it?

*Interviewee*: Well, I’ve been doing that for several years now. You can tell for yourself where you have pain and where you don’t. (P4, 04:52)

#### Medical app

Overall, participants reported easy and intuitive use of the app, even with self-perceived low digital- and language skills.

“I didn’t have any difficulties with that, so that was easy also for me with my language level and my knowledge with these new things, with apps. I’m a little bit old-fashioned, not like my kids for example with the stuff, but I didn’t have to ask my kids for help or something.” (P1, 09:02).

“It’s well operable, in any case. It’s self-explanatory. The questions appear and then you answer them and then there’s just “Submit” at the bottom and then the questionnaire is gone, so that wasn’t a problem.” (P5, 04:33).

Patients reported that the app helps them to better assess their disease condition.

“When I have seen that the curve has actually always been recurrent, so it has never gone beyond the red. I don’t know how to explain it. For me, it was actually like this: Gee, the last four months have actually been relatively stable, except for the one slip. That’s actually a self-confirmation that everything is actually going well.” (P5, 07:22).

However, some of the questions asked (weekly routine assessment of patient index data 3 (RAPID3) questionnaire [24]) were difficult to understand or ambiguous:

“So there were a few questions that I don’t have at home, for example, like turning a faucet. I don’t have that at home, but I answered it for example when I open a bottle.” (P1: 07:02).

“I was a bit confused as to whether running was actually meant or the Franconian term for walking. Or just others, if they somehow go jogging or go running. Then they also say running. In this respect, it must be clarified what is meant there. Important point.” (P3, 05:20).

#### CRP self-sampling

The independent collection of capillary blood was also described by several interview partners as easy, painless and intuitive. In the trial, the blood collection was performed under the supervision of medical staff. Nevertheless, most interviewees reported that they could well imagine performing the blood collection independently at home:

*Interviewer:* “Could you imagine doing that on your own at home without supervision?”

*Interviewee*: “Yes, without any problems. It’s like a COVID test or like being a diabetic. It’s not different, thus I canreally imagine that.” (P15, 06:08)

While other interviewees described challenges in the sampling process:

“But what I found difficult is getting those drops of blood in there into the container. That was totally hard because it kind of doesn’t soak in and there was always this bubble. So I was sort of in the wrong place. I don’t know if I’m the only one, but I found it really hard. Maybe there should be something else…” (P2, 09:45).

#### Joint self-examination

Several patients reported no problems performing the joint self-examination, as for some this was already part of their disease-management routines (P4 - see above). Less experienced patients reported the provided instruction video to be helpful, while one patient felt confused by it:

“So in the meantime on the surface of my hand I can also see it visually, that there are now some bulges, without somehow touching myself, but the elbow is difficult. For example, my hip joint, that hurts me then also, you can’t determine it somehow. And in this video, they even excluded this palm, yeah. My palm hurts sometimes.” (P2, 08:20).

#### Suggestions for improvement

Users made various suggestions to improve the follow-up model, mainly to enhance patient-physician communication, see Table 3.

**Table 3:** Participant suggestions to improve the investigated care-model

“Maybe you could somehow **incorporate a chat**, so if there are questions that you can write in this app and also get an answer. That could perhaps be improved in this app. And of course, revise the questions a little bit regarding opening a bottle, bathtub and…” (P2, 15:21)
“The only thing that might be done, which I now thought: There is always thequestionnaire, the last 7 days, whether you had an attack, a rheumatic attack. It might be good (…) if one could perhaps add something to it. In my case it was only the knee that was affected. And that would be good if you could note that. That wasn’t the case now, so you couldn’t do that. **You could only answer the given questions, but you couldn’t add anything yourself.** (P5, 05:23)
“Would someone who sees my scores… **would a doctor get in touch with me or do I** then have, as before, if the pain is unbearable or if I’m feeling really bad, **just call** and say I can’t wait until the next appointment because I’m not feeling well?” (P10, 12:38)
“You could **include the self-examination instruction video in the app**. You do a little video of someone doing it reasonably. That would be a nudge. If you say, okay, now I’m in pain, did I not examine properly or not? Okay, then I go to the video clip and then I take a look at it, just like on YouTube.“ (P15, 08:32)

### Transfer to standard rheumatology care

**Fig. 2:**
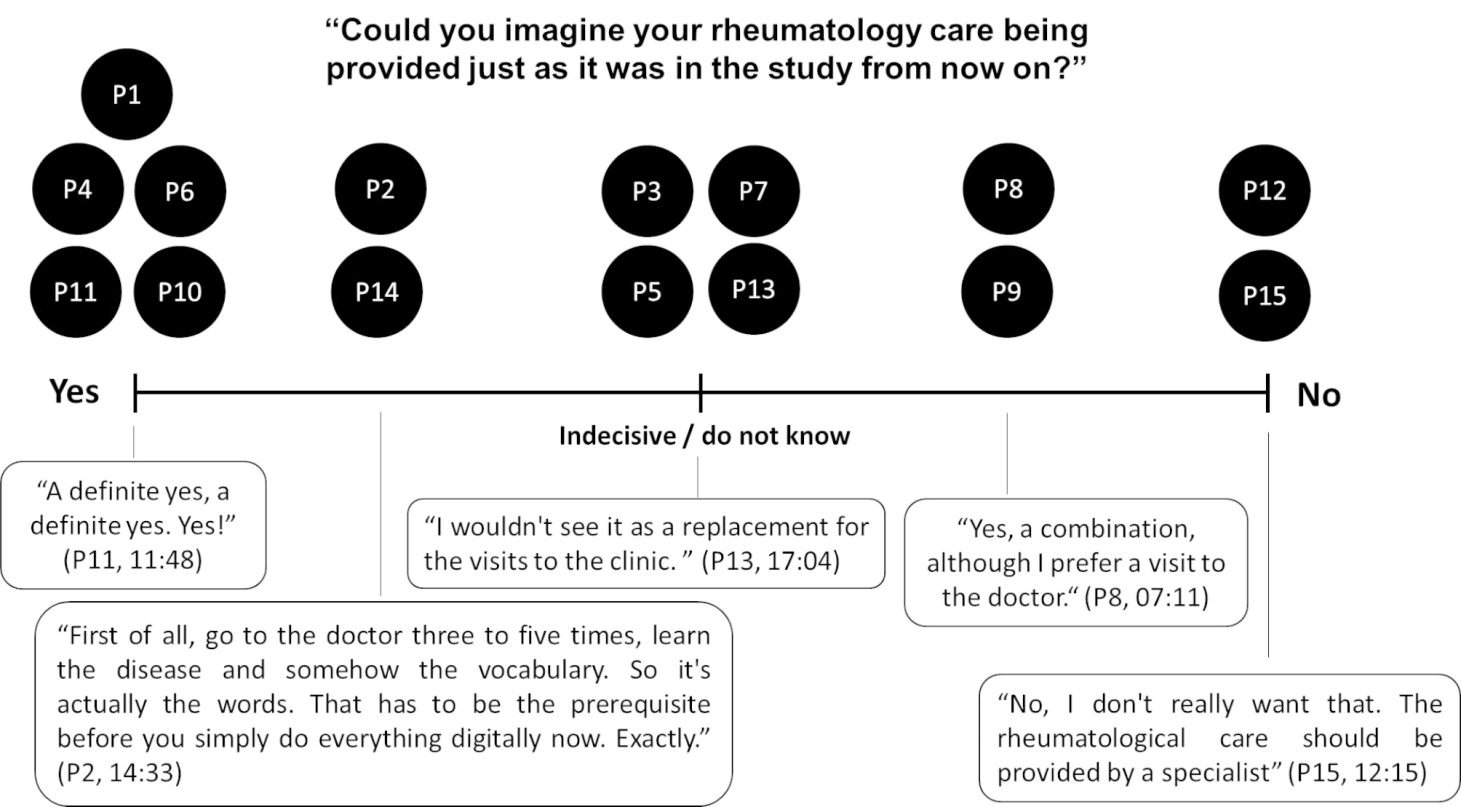
Perspectives on transferability to standard care

In the interviews, we identified a broad spectrum of opinions as to whether, and how, the investigated follow-up model should be transferred into patients’ routine rheumatology care, see Fig. 2. While several trial participants gave a definite affirmative answer or recommended certain disease experience and knowledge as an inclusion criterion; other trial participants were indecisive or suggested combinations of the investigated follow-up model and their standard care. Still, others clearly objected.

#### Opportunities

Trial participants described that the monitoring approach enables them to monitor their disease progression and eventually assess whether medication needs to be adjusted. In addition, the progress curves allow users to relate their own disease state to individual health behavior and lifestyle. The interviewees associated this with increased knowledge on their disease and a more active, controlling role in their disease management:

“So the differences are that I didn’t control myself before: ‘Yes, it hurt, I went to the doctor, the doctor gave me pills and that was it.’ Because of the fact that I participated in this study, I started doing something for myself. I didn’t let it slide, now I really had to check: Is there anything, do you see anything, is something changing? And if that had been the case - thank God it wasn’t - then I could have described it to the doctor during my next visit. If I hadn’t participated in the study, nothing would have changed: It’s just the way it is, it hurts. I would not have dealt with it so intensively. " (P11, 10:48).

Trial participants reported the reduction of appointments and journeys to the rheumatology practice to be time- and money-saving. Furthermore, some interviewees reflected on the lack of rheumatology care resources in Germany.

“Well, first of all, compared to the number of patients there is not enough rheumatology care in Germany. For instance, since I am well adjusted, I do not need a direct contact with the doctor since there is not much to talk about. In other words, if it worked by means of telemedicine, it would be completely sufficient and I think that this is the case for many patients who are not in an acute situation.” (P14, 07:09).

Similarly, a rheumatologist highlighted the new care models’ potential to save resources in rheumatology care:

“I believe that you can replace doctor’s appointments, if you leave aside the whole budgeting and all that, especially if travel distances are long and if patients have simply been in remission for a long time, who were previously seen once a quarter, that you can simply replace these appointments, of course.“ (Focus group, Rheumatologist, 41:44).

#### Barriers

A substantial boundary to the transfer of the investigated care model was the limited personal contact to health care professionals, specifically the treating rheumatologist.

“But the visits to the clinic are very important to me, because then you have the personal conversation with the doctor. At least that is very important to me. Nevertheless, in the meantime, there could always be things like in the study, that you look at it more closely, now maybe weekly or biweekly do some test or palpate joints or whatever. But for me personally, the appointments in the clinic are very important after all.” (P3, 16:52).

This was also discussed by a rheumatology nurse in the focus group.

“I like to talk to people and they like to talk to me, and I think that’s also a very important point in everything we do. People also want to be talked to, they also want to maybe say something about it right and left, which is not just about their swollen wrist. And I believe that the specialist assistants, together with the rheumatologists, are also in a position to include this a little bit in all the progress and in all that we will certainly have to achieve in a few years. For me, it would be important to have a combination of real people and real presence, but of course also digitalization.” (Focus group, Rheumatology Nurse, 01:03:55).

Patients reported experience with the disease, respectively, a high level of knowledge and information on the diseases as essential prerequisites to effectively implement the new care model. For instance, patients have to know certain terms and vocabulary in order to use the medical app and perform joint self-examination. Additionally, the relatively small blood volume, limiting the number of laboratory tests and biomarker insights was described as limitation of the CRP self-sampling, requiring in-person visits in the medical practice. Some trial participants reported the possession of smartphone as well as technical skills to be a barrier:

“It might be related to interest or education, but when you do not possess a smartphone, then you certainly cannot participate.” (P3, 09:50).

In the focus group, rheumatologists reported concerns of ePRO monitoring causing additional demand on already scarce resources, accompanied by financial losses. Furthermore, the outpatient appointment and reimbursement system of ambulatory rheumatology care was addressed and discussed as a barrier that must be overcome in order to successfully implement the new follow-up model into standard care.

“I certainly also see that this could mean significant financial losses for the practices, but here again the question: What would happen if, theoretically, digital monitoring, which is designed differently than it is now, were to provide us with at least as good or perhaps even better monitoring of patients? Would we actually have to change our system and not just say, okay, this doesn’t fit into our quarterly logic? Because that’s part of the study, to break up this quarterly logic in order to see if there aren’t alternatives?” (Focus group, Rheumatologist, 43:03).

#### Potential user groups

According to the trial participants, the investigated follow-up model is suitable for patients in remission with a stable course of the disease. Interviewees reported that the new follow-up requires certain experience with- and knowledge of one’s own disease. Patients need to possess a smartphone and have certain technical skills to participate. Blood collection could be impeded when patients suffer impaired vision. Additionally, interviewees surmised that younger patients might get along better with the investigated follow-up model than older patients.

Regardless of the characteristics of the users, both in the interviews and in the focus group, participants highlighted the relevance of the patients’ freedom to choose the follow-up form.

“But you still have to consider the patient’s right to wish and choose the form of medical treatment. There are people who don’t want to or who can’t monitor themselves in this way for very different reasons, psychological reasons or because they also have other intellectual prerequisites or are in a social situation where that’s not possible or where it’s just a matter of course - we are talking about a marathon - stop any events within the time frame where the patient or patients are no longer able to carry out this self-monitoring.“ (Focus group - Patient Rep., 32:53).

## Discussion

In this study, we explored RA patients’ experiences of a new care model, patients’ and HCP experiences regarding current challenges of rheumatology care, and barriers and opportunities of the new care model. There is a lack of studies examining asynchronous RA monitoring strategies [25]. To our knowledge, this is the first study evaluating a care model in rheumatology where patients use a point-of-care CRP test combined with ePRO and joint self-examination. Overall, the results highlight the urgency for new care models, as all stakeholders expressed serious challenges to the current situation. Workforce shortage was considered as the main cause which is in line with current study results [25]. Due to this lack of professional staff, patients are facing substential diagnostic delays, often causing irreversible damage [26]. In line with other reports, current care was reported to be inefficient and burdensome [27, 28].

Reported patient experiences suggest high usability and acceptance among patients. Most suggested improvements targeted patient-physician communication. Patients wanted to be automatically contacted, have a feature to request a call-back or chat option. Similarly, patients reported the trouble reaching local staff, as during the study we had to use the traditional local communication options. Improving this access to HCP feedback should be a main goal of a new care model approach. Prior studies reported on the benefit of guidebooks [29] and an initial structured training with checklists to inform when to contact the rheumatology team [27].

Rheumatologists are concerned about ePRO monitoring causing additional demand on already scarce resources. Even if ePRO results would only be used for physician visits they could help patients to legitimize their symptoms [30] and improve patient-provider interaction [12]. Decentralization could enable easier access, however, patients seem to prefer communicating with familiar HCP [31]. Delegation of ePRO monitoring to rheumatology nurses, as conducted by de Thurah et al. seems to be a safe option [15, 32]. Automated monitoring of ePROs with predefined notification cut-offs could also facilitate this approach. Interestingly, parts of the validated questionnaire used (RAPID3) seemed outdated or not clear enough for many patients, highlighting the need for continuous reevaluation. A PIFU-based strategy has been demonstrated to be safe, yet previous qualitative research revealed that it also causes patient anxiety [27] due to more patient responsibility [33] and implementation can be facilitated by increasing the responsiveness of the system and the system incorporating regular disease monitoring. Overall, we believe a safety net in form of planned semi-automated asynchronous monitoring provides a balanced risk-benefit ratio [15]. Some patients who might lack confidence, have trouble correctly detecting disease activity or do not want to be a burden could be negatively affected by a PIFU system [34]. Müskens et al. recently reported that a Dutch RA telehealth self-management platform was used selectively, by rather young and highly educated patients, potentially widening already existing social health inequalities [35]. In this sense, our results show that patients and stakeholders consider the new care model a useful alternative to standard RA-care, yet, it does not represent a one fits all approach, as it might offer risks for not-suitable patients and potential for exclusion of certain patient groups. In the worst case, the level of self-management in RA follow-up may be too high, resulting in no or inaccurate ePROs, test and self-performed examination results, resulting in reduced quality of care or in patients not receiving RA therapy at all. Thus, we compiled a brief checklist that may guide HCPs in the first assessment of patient suitability to the trialled care model **(**Table 4**)**.

**Table 4:** Checklist for assessing appropriateness of digitally-supported patient-centered follow-up

Favorable Factors	Unfavorable Factors
+ Consent to participate in flexible digitally-supported patient-centered follow-up	+ No Consent to participate in flexible digitally-supported patient-centered follow-up
+ Patients in remission	+ Patients in an acute situation
+ High health literacy and disease knowledge	+ Low health literacy and missing disease knowledge
+ Smartphone, technical skills	+ No smartphone, low technical skills
+ Disease with serological biomarker(s) relevant for disease monitoring (systemic lupus erythematosus, vasculitis, rheumatoid arthritis, spondyloarthritis, psoriatic arthritis, autoinflammatory diseases)	+ No clinically relevant serological bio-marker available (i.e. osteoarthritis)
+ Analysis of few biomarkers sufficient (i.e. CRP, anti-dsDNA)	+ Under anticoagulant therapy
+ Long travel / queue time for venous blood collection	+ Poor wound healing, eczema and other inflammatory skin disease
+ Limited availability due to work/family responsibilities	+ Reduced finger strength/function and pain
+ Immobile patient (high effort/burden associated with medical consultation)	+ Impaired vision
+ High patient adherence	+ Previous failure of blood self-collection
+ Longer disease duration	+ No interest in self-collection

In line with previous work [28], rheumatologists also expressed concerns about abandoning pre-scheduled visits with “easy” remission, and potentially ending up with nothing but complicated cases. This would also cause financial concerns, as rheumatologists are currently not reimbursed for ePRO monitoring and are compensated with the same amount, irrespective of how complex a patient is. Fewer office visits of patients in remission would reduce net reimbursement, causing clear financial disincentives. Value-based healthcare should ultimately be targeted to prevent this misdevelopment and false incentives.

Experienced patients felt highly confident examining their joints, yet rheumatologists questioned the reliability of self-performed joint counts. Radner et al. demonstrated that self-performed joint counts are more reliable in patients in remission [36]. A training session did not improve performance. Digital biomarkers could enable objective evaluation or even flare prediction [37, 38].

Due to the COVID-19 pandemic, patients were already used to perform POC tests. The value of at-home POC measurements has previously been demonstrated by fecal calprotectin in patients with inflammatory bowel disease. Recently, Riches et al. demonstrated that usage of an app and urate self-testing substantially improved attainment of urate targets compared with usual care [39]. We believe that adding POC CRP tests to remote RA monitoring may add an important objective cornerstone. Additionally, we believe that enabling results within minutes (POC) is crucial. We chose a semi-quantitative test as a compromise of accuracy and availability. Costly professional POC machines would enable exact results, mailing to a laboratory would be less expensive but cost too much time. RA patients seem to prefer upper-arm self-sampling compared to conventional finger-pricking [17].

This study has some limitations to consider. All patients were recruited from one study site, so the results might not be generalisable. Secondly, participating rheumatologists did not have access to any preliminary results of the trial and did only describe their individual study experiences. Recall bias cannot be excluded, e.g. due to the time difference between self-sampling and the interview. In addition, results may be biased toward the benefits as participants agreed to participate in the study first hand. Further biases are possible due the data being collected via phone call or video conference: loss of non-verbal communication, distractions, lack of confidential atmosphere, and thus social-desiarability bias. Specifically, in the focus groups, originally intented to discursevely contrast opinions among professional groups, we observed a very homogenous opinion profile, which might also be due to hierarchy dynamics between the included professions and individuals. Futhermor, since this study essentially examines the implementation of complex health interventions, another limitation could be the inductive analysis approach and the absence of an implementation framework (such as Consolidated Framework for Implementation Research (CFIR)). A major strength of this study is the patient involvement, the diversity of the included patients (age, disease duration, sex) and staff (nurses, university- and practice-based rheumatologists).

## Conclusion

Our results highlight current challenges in rheumatology and the urgent need for a transformation of care enabling more flexibility and need-adapted visits. The investigated model was easy to use and well accepted among patients. Yet, many ways of improvement were suggested and multiple barriers could be identified.

## Electronic supplementary material

Below is the link to the electronic supplementary material.

Supplementary Material 1

Supplementary Material 2

Supplementary Material 3

## Data Availability

All data relevant to the study are included in the article or uploaded as supplementary material. For further questions regarding the reuse of data, please contact the corresponding author (felix.muehlensiepen@mhb-fontane.de).
