# Supplementary material for: Digitally-supported patient-centered asynchronous outpatient follow-up in rheumatoid arthritis - an explorative qualitative study
Source: BMC Health Serv Res. 2022 Oct 28;22:1297. doi: 10.1186/s12913-022-08619-6 (PMC9614742; doi:10.1186/s12913-022-08619-6)
Supplement: Supplementary file 1 — Supplementary Material 1 [file 12913_2022_8619_MOESM1_ESM.docx]

**Supplemental Material 1. Interview Guide**

| **Guiding Questions** | **Check Aspects** |
| --- | --- |
| Could you please describe your standard RA follow-up care? | Standard rheumatology care, challenges, user experiences |
| You have been participating in the TELERA study for a few weeks now.  Could you please tell me what happened in the study?  How did you experience each component of the study? | CRP self-sampling, medical app, joint self-examination |
| How did the rheumatology care in the study differ from your standard care?  Compared to your standard care, were there any benefits?  Were there any drawbacks? | Differences to standard RA follow-up, benefits, drawbacks |
| Could you imagine your rheumatology care being provided just as it was in the study from now on? Please describe. | Transferability to standard care |
