# Supplementary material for: Digitally-supported patient-centered asynchronous outpatient follow-up in rheumatoid arthritis - an explorative qualitative study
Source: BMC Health Serv Res. 2022 Oct 28;22:1297. doi: 10.1186/s12913-022-08619-6 (PMC9614742; doi:10.1186/s12913-022-08619-6)
Supplement: Supplementary file 2 — Supplementary Material 2 [file 12913_2022_8619_MOESM2_ESM.docx]

**Supplemental Material 2. Coding Tree**

| **Main Category** | **Category** | **Distinction / Scaling** | **Code (Examples)** |
| --- | --- | --- | --- |
| Standard RA follow-up care | Challenges | Patient | Quarterly visits, long distances to the rheumatologist, workforce shortage |
|  |  | HCP | Quarterly visits, long waiting times, workforce shortage, not efficient |
| TELERA trial user experiences | Medical app | Positive | Easy usage, improved disease monitoring, high success rate |
|  |  | Negative | Questions ambiguous, lack of feedback |
|  | CRP self-sampling | Positive | Smooth application, easy, painless |
|  |  | Negative | Limited scope of test due to low blood volume, challenges during sampling process |
|  | Joint self-examination | Positive | Part of patient routines, video helpful |
|  |  | Negative | Video confusing, difficulties in detecting abnormalities |
|  | Suggestions for improvement | / | Improved patient-physician communication |
| Transfer to standard rheumatology care | Opportunities | Patient | Empowerment, time savings in health care delivery, improved disease monitoring independence, flexibility |
|  |  | HCP | Time savings in health care delivery, access to patient data |
|  | Barriers | Patient | Limited personal contact, limitations of the CRP self-sampling |
|  |  | HCP | Limited personal contact, time consuming, financial losses |
|  | Potential user groups | Favorable | Patients in remission, high health literacy and disease knowledge, smartphone, technical skills |
|  |  | Unfavorable | Patients in acute situation, low health literacy and disease knowledge, no smartphone, lack technical skills |
